# Estimating Quantum Mutual Information of Continuous-Variable Quantum States by Measuring Purity and Covariance Matrix

**DOI:** 10.3390/e24070940

**Published:** 2022-07-06

**Authors:** Jiyong Park

**Affiliations:** School of Basic Sciences, Hanbat National University, Daejeon 34158, Korea; jiyong.park@hanbat.ac.kr

**Keywords:** quantum mutual information, purity, covariance matrix

## Abstract

We derive accessible upper and lower bounds for continuous-variable (CV) quantum states on quantum mutual information. The derivations are based on the observation that some functions of purities bound the difference between quantum mutual information of a quantum state and its Gaussian reference. The bounds are efficiently obtainable by measuring purities and the covariance matrix without multimode quantum state reconstruction. We extend our approach to the upper and lower bounds for the quantum total correlation of CV multimode quantum states. Furthermore, we investigate the relations of the bounds for the quantum mutual information with the bounds for the quantum conditional entropy.

## 1. Introduction

Quantum mutual information quantifies the total correlation in a quantum state. It provides a valuable tool for investigating various quantum phenomena, e.g., area laws in quantum systems [1,2], quantum phase transitions [3,4] and quantum thermodynamics [5,6]. Furthermore, quantum mutual information also plays critical roles in quantifying quantum correlations, e.g., squashed quantum entanglement [7] and quantum discord [8], and assessing the performance of quantum information protocols [9].

To obtain quantum mutual information of a quantum state, we need to perform multimode quantum state tomography, i.e., a standard method for inferring quantum states [10]. However, the amount of resources required for quantum state tomography is large and radically increased by the number of modes. Furthermore, calculating quantum mutual information of a continuous variable (CV) quantum state is often computationally challenging because of the requirement of solving infinite-dimensional eigenvalue problems [11]. It is thus favorable if efficient and reliable estimation methods exist for the quantum mutual information. The classical correlation attainable by local positive operator-valued measures (POVMs) is a known lower bound for quantum mutual information [12]. However, it is generally difficult to find and realize the optimal local POVMs to extract the maximum classical correlation from CV quantum states [13].

On the other hand, the extremality of Gaussian states [14] has attracted considerable research interest in CV quantum information [15,16]. A measure M[ρ] has Gaussian extremality if it is always lower-bounded, i.e., $M\left[\rho \right]\geq M\left[\rho_{G}\right]$, or upper-bounded, i.e., $M\left[\rho \right]\leq M\left[\rho_{G}\right]$, by its Gaussian reference $\rho_{G}$ having the same first-order and second-order quadrature moments with the state ρ. A Gaussian state is uniquely determined by its first-order and second-order quadrature moments. Therefore, if a measure has Gaussian extremality, the measure can be efficiently estimated by homodyne detection. In the same vein, one may prefer if the quantum mutual information has Gaussian extremality [17]. However, quantum mutual information does not exhibit Gaussian extremality [18].

Fortunately, the bounds for the difference in the quantum mutual informations of a quantum state and its Gaussian reference are determinable. In this study, we derive the upper and lower bounds for the quantum mutual information of a quantum state by adding some functions of purities to the quantum mutual information of its Gaussian reference. The bounds are efficiently obtainable by measuring purities and the covariance matrix without multimode quantum state reconstruction. We generalize the proposed approach to obtain the upper and lower bounds for the quantum total correlation of CV multimode quantum states. We also investigate how the bounds for the quantum mutual information are related to the bounds for the quantum conditional entropy.

## 2. Preliminaries

Quantum mutual information of a two-mode quantum state $\rho_{12}$ is defined as quantum relative entropy of the global quantum state $\rho_{12}$ with respect to the product form of local quantum states $\rho_{1}⊗\rho_{2}$,

(1) $$I\left(\rho_{12}\right)=S(\rho_{12}\left|\right|\rho_{1}⊗\rho_{2})=S\left(\rho_{1}\right)+S\left(\rho_{2}\right)-S\left(\rho_{12}\right),$$

where S(τ||σ)=tr[τlnτ−τlnσ] denotes the quantum relative entropy of τ with respect to σ and S(τ)=−tr[τlnτ] is the von Neumann entropy of τ. Throughout the paper, we employ the natural unit of information, i.e., nat, instead of the binary unit of information, i.e., bit, to quantify the amount of information. Note that the quantum mutual information of a quantum state ρ is relevant to the minimum amount of noise required for erasing the correlation of the quantum state ρ [19] and the maximally achievable secure communication rate between the modes of the quantum state ρ [20].

The quantum Rényi entropy of a quantum state ρ is defined as follows:

(2) $$S_{\alpha }\left(\rho \right)=\frac{1}{1-\alpha }\ln\mathrm{tr}\rho^{\alpha }.$$

Note that the equation becomes identical to the von Neumann entropy in the limit of α=1, i.e., $\lim_{\alpha \to 1}S_{\alpha }\left(\rho \right)=S\left(\rho \right)$, and it is directly related to the purity $\mu_{\rho }\equiv \mathrm{tr}\rho^{2}$ when α=2, i.e., $S_{2}\left(\rho \right)=-\ln\mu_{\rho }$.

An *N*-mode Gaussian state $\rho_{G}$ is uniquely determined by its mean values of local quadrature operators, i.e., ${\langle \hat{Q}\rangle }_{\rho_{G}}={\left\{{\langle {\hat{q}}_{1}\rangle }_{\rho_{G}},{\langle {\hat{p}}_{1}\rangle }_{\rho_{G}},\ldots ,{\langle {\hat{q}}_{N}\rangle }_{\rho_{G}},{\langle {\hat{p}}_{N}\rangle }_{\rho_{G}}\right\}}^{T}$ with ${\langle \hat{O}\rangle }_{\rho }=\mathrm{tr}\left(\rho \hat{O}\right)$, and its covariance matrix $\Gamma_{\rho_{G}}$ whose elements are defined as follows:

(3) $$\Gamma_{\rho_{G},jk}=\frac{1}{2}{\langle {\hat{Q}}_{j}{\hat{Q}}_{k}+{\hat{Q}}_{k}{\hat{Q}}_{j}\rangle }_{\rho_{G}}-{\langle {\hat{Q}}_{j}\rangle }_{\rho_{G}}{\langle {\hat{Q}}_{k}\rangle }_{\rho_{G}},$$

where ${\hat{q}}_{j}=\frac{1}{\sqrt{2}}\left({\hat{a}}_{j}+{\hat{a}}_{j}^{†}\right)$ and ${\hat{p}}_{j}=\frac{1}{i\sqrt{2}}\left({\hat{a}}_{j}-{\hat{a}}_{j}^{†}\right)$ are the position and momentum operators for *j*th mode, respectively.

For the Gaussian state $\rho_{G}$, the von Neumman entropy $S(\rho_{G})$ and the quantum Rényi-2 entropy $S_{2}\left(\rho_{G}\right)$ are expressed as follows:

(4) $$S\left(\rho_{G}\right)=\sum_{j=1}^{N}g\left(\lambda_{j}\right),$$

with $g\left(x\right)=\left(x+\frac{1}{2}\right)\ln\left(x+\frac{1}{2}\right)-\left(x-\frac{1}{2}\right)\ln\left(x-\frac{1}{2}\right)$ and

(5) $$S_{2}\left(\rho_{G}\right)=\ln\left(2^{N}\sqrt{\det\Gamma_{\rho_{G}}}\right)=\sum_{j=1}^{N}\ln\left(2\lambda_{j}\right),$$

respectively, where $\lambda_{j}$ with j∈{1,2,…,N} denotes the *j*th symplectic eigenvalue of the covariance matrix $\Gamma_{\rho_{G}}$ [16].

One may employ local POVMs and classical communications to extract the correlation from a two-mode quantum state. Throughout the paper, we refer to the mutual information of the joint probability distribution obtained by local POVMs as the classical mutual information extractable by joint local POVMs. Here we express a set of joint local POVMs as $\left\{\hat{\Pi }\right\}=\left\{{\hat{\Pi }}_{j}^{\left(1\right)}⊗{\hat{\Pi }}_{k}^{\left(2\right)}\right\}$ with $\sum_{j}{\hat{\Pi }}_{j}^{\left(1\right)}=\sum_{k}{\hat{\Pi }}_{k}^{\left(2\right)}=I$. The classical mutual information of a two-mode quantum state ρ extractable by the joint local POVMs is obtained as follows:

(6) $$C_{\left\{\hat{\Pi }\right\}}\left(\rho \right)=D_{\mathrm{KL}}(P_{12}\left(z_{1},z_{2}\right)\left|\right|P_{1}\left(z_{1}\right)P_{2}\left(z_{2}\right)),$$

where $D_{\mathrm{KL}}\left(X||Y\right)=\sum_{z}X\left(z\right)\left[\ln X\left(z\right)-\ln Y\left(z\right)\right]$ is the Kullback–Leibler divergence [21] between the two probability distributions *X* and *Y*, $P_{12}\left(j,k\right)=\mathrm{tr}\left[\rho \left({\hat{\Pi }}_{j}^{\left(1\right)}⊗{\hat{\Pi }}_{k}^{\left(2\right)}\right)\right]$ is the probability to obtain the *j*th outcome on the first mode and the *k*th outcome on the second mode, and $P_{n}\left(j\right)=\mathrm{tr}\left[\rho_{n}{\hat{\Pi }}_{j}^{\left(n\right)}\right]$ with n∈{1,2} is the probability to obtain the *j*th outcome on the *n*th mode. It is known that $C_{\left\{\Pi \right\}}\left(\rho \right)$ is upper-bounded by $\min\{S\left(\rho_{1}\right),S\left(\rho_{2}\right),I\left(\rho \right)\}$ and $C_{\left\{\hat{\Pi }\right\}}\left(\rho \right)=I\left(\rho \right)$ can be achieved for classically correlated states [12]. In addition, if $I\left(\rho \right)>\min\{S\left(\rho_{1}\right),S\left(\rho_{2}\right)\}$ is satisfied, $C_{\left\{\hat{\Pi }\right\}}\left(\rho \right)$ is strictly smaller than I(ρ) for any set of joint local POVMs. Finding the optimal set of local POVMs to maximize $C_{\left\{\hat{\Pi }\right\}}\left(\rho \right)$ is generally difficult for high-dimensional quantum states. For this reason, we focus on homodyne detection, which is experimentally feasible for CV quantum states.

The classical mutual information extractable by joint homodynde detection is expressed as follows:

(7) $$C_{\mathrm{HD}}^{\left\{\phi_{1},\phi_{2}\right\}}\left(\rho \right)=D_{\mathrm{KL}}(P_{\rho }^{\left\{\phi_{1},\phi_{2}\right\}}\left(q_{1},q_{2}\right)\left|\right|P_{\rho_{1}⊗\rho_{2}}^{\left\{\phi_{1},\phi_{2}\right\}}\left(q_{1},q_{2}\right)),$$

where $\phi_{j}$ denotes the phase angle for the homodyne detection on the *j*th mode. Here the joint probability distribution $P_{\rho }^{\left\{\phi_{1},\phi_{2}\right\}}\left(q_{1},q_{2}\right)$ is obtained by the following expression:

(8) $$P_{\rho }^{\left\{\phi_{1},\phi_{2}\right\}}\left(x_{1},x_{2}\right)=\mathrm{tr}(\rho \overset{2}{\underset{j=1}{⨂}}|q_{j,\phi_{j}}=x_{j}\rangle \langle q_{j,\phi_{j}}=x_{j}|),$$

where $|q_{j,\phi_{j}}=x_{j}\rangle$ is the unnormalized eigenstate of the rotated local quadrature operator ${\hat{q}}_{j,\phi_{j}}={\hat{q}}_{j}\cos\phi_{j}+{\hat{p}}_{j}\sin\phi_{j}$ for the *j*th mode with the eigenvalue $x_{j}$, i.e., ${\hat{q}}_{j,\phi_{j}}|q_{j,\phi_{j}}=x_{j}\rangle =x_{j}|q_{j,\phi_{j}}=x_{j}\rangle$. We define $C_{\mathrm{HD}}\left(\rho \right)$ as the maximum of $C_{\mathrm{HD}}^{\left\{\phi_{1},\phi_{2}\right\}}\left(\rho \right)$ optimized over the phase angles:

(9) $$C_{\mathrm{HD}}\left(\rho \right)\equiv \max_{\phi_{1},\phi_{2}}C_{\mathrm{HD}}^{\left\{\phi_{1},\phi_{2}\right\}}\left(\rho \right).$$

## 3. Bounds for Quantum Mutual Information

Our upper and lower bounds on quantum mutual information of a two-mode quantum state ρ are expressed as follows:

(10) $$I_{+}\left(\rho \right)\equiv I\left(\rho_{G}\right)+F\left(\rho \right),$$

and

(11) $$I_{-}\left(\rho \right)\equiv I\left(\rho_{G}\right)-\min_{j\in \{1,2\}}F\left(\rho_{j}\right),$$

respectively, where the function F(σ) for an *N*-mode quantum state σ is expressed as follows:

(12) $$F\left(\sigma \right)=S_{2}\left(\sigma_{G}\right)-S_{2}\left(\sigma \right)+N\ln\frac{e}{2},$$

where $S_{2}\left(\sigma \right)$ and $S_{2}\left(\sigma_{G}\right)$ are directly related to the purities of the *N*-mode quantum state σ and its Gaussian reference $\sigma_{G}$, respectively. Here $I_{+}\left(\rho \right)$ is always non-negative because I(σ) and F(σ) are non-negative for an arbitrary quantum state σ. However, $I_{-}\left(\rho \right)$ can be negative when $\min_{j}F\left(\rho_{j}\right)$ is greater than $I(\rho_{G})$, which signifies that $I_{-}\left(\rho \right)$ is useful when $I(\rho_{G})$ is sufficiently large.

We can derive Equation (10) by using $S\left(\sigma_{G}\right)\geq S\left(\sigma \right)$ [22] and the inequality appeared in [23]:

(13) $$S_{2}\left(\sigma_{G}\right)-S_{2}\left(\sigma \right)\geq S\left(\sigma_{G}\right)-S\left(\sigma \right)+N\ln\frac{2}{e},$$

for an *N*-mode quantum state σ. The proof is as follows:

(14) $$\begin{array}{cc} I(\rho ) & =S\left(\rho_{1}\right)+S\left(\rho_{2}\right)-S\left(\rho \right)\\ \; & \leq S\left(\rho_{1,G}\right)+S\left(\rho_{2,G}\right)-S\left(\rho \right)\\ \; & \leq S\left(\rho_{1,G}\right)+S\left(\rho_{2,G}\right)-S\left(\rho_{G}\right)+F\left(\rho \right)\\ \; & =I\left(\rho_{G}\right)+F\left(\rho \right)\\ \; & =I_{+}\left(\rho \right). \end{array}$$

We can derive Equation (11) by using the fact that the non-Gaussian measure by quantum relative entropy is non-increasing under partial trace [17]:

(15) $$S\left(\rho_{G}\right)-S\left(\rho \right)\geq S\left(\rho_{j,G}\right)-S\left(\rho_{j}\right),$$

and Equation (13). The proof is as follows:

(16) $$\begin{array}{cc} I(\rho ) & =S\left(\rho_{1}\right)+S\left(\rho_{2}\right)-S\left(\rho \right)\\ \; & \geq \max_{j,k\in \{1,2\}}\left\{S\left(\rho_{j,G}\right)-S\left(\rho_{G}\right)+S\left(\rho_{k\neq j}\right)\right\}\\ \; & \geq \max_{j,k\in \{1,2\}}\left\{S\left(\rho_{j,G}\right)-S\left(\rho_{G}\right)+S\left(\rho_{k\neq j,G}\right)-F\left(\rho_{k\neq j}\right)\right\}\\ \; & =I\left(\rho_{G}\right)-\min_{j\in \{1,2\}}F\left(\rho_{j}\right)\\ \; & =I_{-}\left(\rho \right). \end{array}$$

We explain how the bounds are accessible without multimode quantum state reconstruction. We first obtain the covariance matrix of the quantum state ρ that can be efficiently measurable through homodyne detection. By using the covariance matrix, we obtain the quantum mutual information $I(\rho_{G})$ and the quantum Rényi-2 entropies of the reference Gaussian state $\rho_{G}$, i.e., $S_{2}\left(\rho_{1,G}\right)$, $S_{2}\left(\rho_{2,G}\right)$, and $S_{2}\left(\rho_{G}\right)$. Finally, the quantum Rényi-2 entropies of the state ρ, i.e., $S_{2}\left(\rho_{1}\right)$, $S_{2}\left(\rho_{2}\right)$, and $S_{2}\left(\rho \right)$, can be obtained without quantum state reconstruction by homodyne detection [24] or parity detection [25].

### 3.1. Gaussian State

If a quantum state σ is Gaussian, its Gaussian reference $\sigma_{G}$ is the quantum state σ itself, i.e., $\sigma_{G}=\sigma$. In addition, a local mode of a multimode Gaussian state is represented by a single-mode Gaussian state. Therefore, it is straightforward to observe that, for every two-mode Gaussian state ρ, $I_{-}\left(\rho \right)$ is smaller than I(ρ) by ln(e/2)≃0.307,

(17) $$I\left(\rho \right)-I_{-}\left(\rho \right)=\ln\frac{e}{2},$$

and $I_{+}\left(\rho \right)$ is greater than I(ρ) by 2ln(e/2)≃0.614,

(18) $$I_{+}\left(\rho \right)-I\left(\rho \right)=2\ln\frac{e}{2}.$$

These results signify that $I_{-}\left(\rho \right)$ and $I_{+}\left(\rho \right)$ work well for a broad range of Gaussian states with small differences.

Here we examine a two-mode squeezed thermal state in the form of $\rho =\hat{S}\left(r\right)\left(\tau_{\bar{n},1}⊗\tau_{\bar{n},2}\right){\hat{S}}^{†}\left(r\right)$ where $\hat{S}\left(r\right)=e^{r({\hat{a}}_{1}^{†}{\hat{a}}_{2}^{†}-{\hat{a}}_{1}{\hat{a}}_{2})}$ is the squeezing operator with squeezing parameter *r* and $\tau_{\bar{n}}=\sum_{k=0}^{\infty }\frac{{\bar{n}}^{k}}{{\left(\bar{n}+1\right)}^{k+1}}\left|k\rangle \langle k\right|$ is the thermal state with the mean photon number $\bar{n}$. Its covariance matrix $\Gamma_{\rho }$ is expressed as follows:

(19) $$\Gamma_{\rho }=\left(\begin{array}{cccc} a & 0 & b & 0\\ 0 & a & 0 & -b\\ b & 0 & a & 0\\ 0 & -b & 0 & a \end{array}\right),$$

with $a=(\bar{n}+\frac{1}{2})\cosh2r$ and $b=(\bar{n}+\frac{1}{2})\sinh2r$. The quantum mutual information of the two-mode squeezed thermal state is obtained by the following expression:

(20) $$I\left(\rho \right)=I\left(\rho_{G}\right)=2g\left(a\right)-2g\left(\sqrt{a^{2}-b^{2}}\right).$$

We have $S_{2}\left(\rho \right)=S_{2}\left(\rho_{G}\right)=-2\ln\left(2\sqrt{a^{2}-b^{2}}\right)$ and

(21) $$S_{2}\left(\rho_{j}\right)=S_{2}\left(\rho_{j,G}\right)=-\ln2a,$$

for j∈{1,2}.

The joint quadrature distribution of the two-mode squeezed thermal state is expressed as follows:

(22) $$P_{\rho }^{\left\{\phi_{1},\phi_{2}\right\}}\left(q_{1},q_{2}\right)=\frac{1}{2\pi \sqrt{u^{2}-v^{2}}}\exp\left[-\frac{u\left(q_{1}^{2}+q_{2}^{2}\right)-2vq_{1}q_{2}}{2(u^{2}-v^{2})}\right],$$

with $u=(\bar{n}+\frac{1}{2})\cosh2r$ and $v=\left(\bar{n}+\frac{1}{2}\right)\sinh2r\cos\left(\phi_{1}+\phi_{2}\right)$. We analytically determine that $C_{\mathrm{HD}}\left(\rho \right)=\ln\frac{a}{\sqrt{a^{2}-b^{2}}}$ is achieved when $\phi_{1}+\phi_{2}=0$.

**Figure 1:**
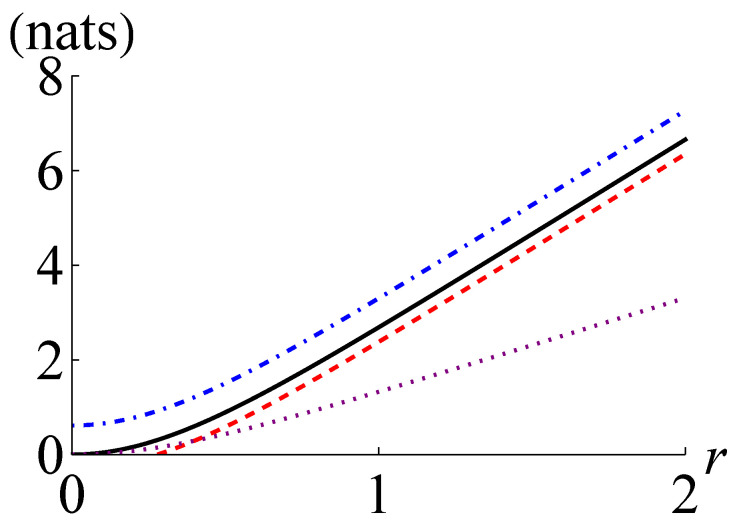
Quantum mutual information I(ρ) (black solid line), its lower and upper bounds $I_{-}\left(\rho \right)$ (red dashed line), and $I_{+}\left(\rho \right)$ (blue dot-dashed line), respectively, and the maximum of the classical mutual information extractable by joint homodyne detection $C_{\mathrm{HD}}\left(\rho \right)$ (purple dotted line) for the two-mode squeezed thermal state with $\bar{n}=1$ against the squeezing parameter *r*. Note that one nat is equal to $\frac{1}{\ln2}≃1.44$ bits.

In Figure 1, we plot I(ρ), $I_{+}\left(\rho \right)$, $I_{-}\left(\rho \right)$ and $C_{\mathrm{HD}}\left(\rho \right)$ for a two-mode squeezed thermal state with $\bar{n}=1$. The curves in Figure 1 show that $I_{-}\left(\rho \right)$ becomes positive for r>0.279 and $I_{-}\left(\rho \right)>C_{\mathrm{HD}}\left(\rho \right)$ happens for r>0.399. Here, $I_{-}\left(\rho \right)$ outperforms $C_{\mathrm{HD}}\left(\rho \right)$ for a wide range of parameters. Interestingly, as the squeezing parameter *r* approaches infinity, the ratio of $I_{-}\left(\rho \right)$ to I(ρ) and the ratio of $I_{+}\left(\rho \right)$ to I(ρ) get closer and closer to unity. This observation manifests that $I_{-}\left(\rho \right)$ and $I_{+}\left(\rho \right)$ work better for Gaussian states with larger quantum mutual information.

### 3.2. Pair Coherent State

We here investigate pair coherent states [26,27] in the form of $|\varphi_{x}\rangle =\frac{1}{\sqrt{I_{0}\left(2x\right)}}\sum_{k=0}^{\infty }\frac{x^{k}}{k!}{|k\rangle }_{1}{|k\rangle }_{2}$ where $I_{n}\left(x\right)$ denotes the modified Bessel function of the first kind of order *n* [28]. Its covariance matrix is expressed as follows:

(23) $$\Gamma_{|\varphi_{x}\rangle \langle \varphi_{x}|}=\left(\begin{array}{cccc} a & 0 & b & 0\\ 0 & a & 0 & -b\\ b & 0 & a & 0\\ 0 & -b & 0 & a \end{array}\right),$$

with $a=\frac{1}{2}+\frac{xI_{1}\left(2x\right)}{I_{0}\left(2x\right)}$ and b=x. The quantum mutual information of the pair coherent state $\rho =|\varphi_{x}\rangle \langle \varphi_{x}|$ is obtained by the following expression:

(24) $$I\left(\rho \right)=-2\sum_{k=0}^{\infty }\frac{x^{2k}}{{\left(k!\right)}^{2}I_{0}\left(2x\right)}\ln\frac{x^{2k}}{{\left(k!\right)}^{2}I_{0}\left(2x\right)}.$$

We have $S_{2}\left(\rho \right)=0$ and

(25) $$S_{2}\left(\rho_{1}\right)=S_{2}\left(\rho_{2}\right)=-\ln\sum_{k=0}^{\infty }{\left\{\frac{x^{2k}}{{\left(k!\right)}^{2}I_{0}\left(2x\right)}\right\}}^{2}.$$

The joint quadrature distribution of the pair coherent state is expressed as follows:

(26) $$P_{\rho }^{\left\{\phi_{1},\phi_{2}\right\}}\left(q_{1},q_{2}\right)=\frac{1}{I_{0}\left(2x\right)}\sum_{j=0}^{\infty }\sum_{k=0}^{\infty }\frac{x^{j+k}}{j!k!}P_{\left|j\rangle \langle k\right|}^{\phi_{1}}\left(q_{1}\right)P_{\left|j\rangle \langle k\right|}^{\phi_{2}}\left(q_{2}\right),$$

where the local quadrature distribution $P_{\left|j\rangle \langle k\right|}^{\phi }\left(q\right)$ in Fock basis is determined by

(27) $$P_{\left|j\rangle \langle k\right|}^{\phi }\left(q\right)=\sqrt{\frac{1}{\pi 2^{j+k}j!k!}}e^{-q^{2}}e^{-i(j-k)\phi }H_{j}\left(q\right)H_{k}\left(q\right),$$

and $H_{n}\left(x\right)$ denotes the Hermite polynomial of order *n* [28]. We numerically determine that the classical mutual information extractable by joint homodyne detection $C_{\mathrm{HD}}^{\left\{\phi_{1},\phi_{2}\right\}}\left(\rho \right)$ is maximized when $\phi_{1}+\phi_{2}=0$.

**Figure 2:**
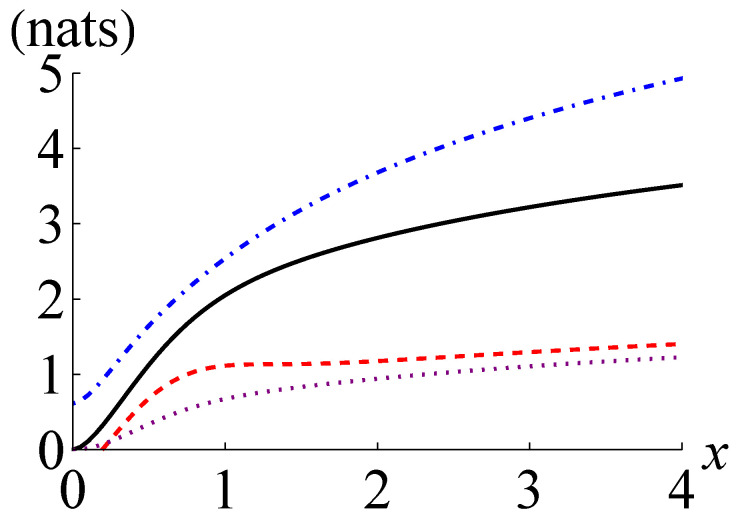
Quantum mutual information I(ρ) (black solid line), its lower and upper bounds $I_{-}\left(\rho \right)$ (red dashed line), and $I_{+}\left(\rho \right)$ (blue dot-dashed line), respectively, and the maximum of the classical mutual information extractable by joint homodyne detection $C_{\mathrm{HD}}\left(\rho \right)$ (purple dotted line) for the pair coherent state $\rho =|\varphi_{x}\rangle \langle \varphi_{x}|$ against the parameter *x*.

In Figure 2, we plot I(ρ), $I_{+}\left(\rho \right)$, $I_{-}\left(\rho \right)$ and $C_{\mathrm{HD}}\left(\rho \right)$ for a pair coherent state $\rho =|\varphi_{x}\rangle \langle \varphi_{x}|$. The curves in Figure 2 reveal that $I_{-}\left(\rho \right)$ becomes positive for x>0.193 and outperforms $C_{\mathrm{HD}}\left(\rho \right)$ for x>0.235. The ratio of $I_{-}\left(\rho \right)$ to I(ρ) has the maximum of about 0.614 at x≃0.60 and the ratio of $I_{+}\left(\rho \right)$ to I(ρ) has and the minimum of about 1.238 at x≃1.05. This observation suggests that $I_{-}\left(\rho \right)$ and $I_{+}\left(\rho \right)$ may generally work better for Gaussian states than non-Gaussian states.

### 3.3. CV Werner State

We here investigate CV Werner state [29,30] in the form of $\rho =f|\psi_{r}\rangle \langle \psi_{r}\left|+\left(1-f\right)|0\rangle \langle 0|⊗|0\rangle \langle 0\right|$ where $|\psi_{r}\rangle =\mathrm{sech}r\sum_{k=0}^{\infty }\tanh^{k}{r|k\rangle }_{1}{|k\rangle }_{2}$ denotes the two-mode squeezed vacuum with the squeezing parameter *r*. Its covariance matrix is expressed as follows:

(28) $$\Gamma_{\rho }=\left(\begin{array}{cccc} a & 0 & b & 0\\ 0 & a & 0 & -b\\ b & 0 & a & 0\\ 0 & -b & 0 & a \end{array}\right),$$

with $a=\frac{1}{2}f\cosh2r+\frac{1}{2}\left(1-f\right)$ and $b=\frac{1}{2}f\sinh2r$. The quantum mutual information of the CV Werner state is obtained by the following expression:

(29) $$\begin{array}{cc} I(\rho ) & =S\left(\rho_{1}\right)+S\left(\rho_{2}\right)-S\left(\rho \right), \end{array}$$

where the von Neumann entropy of *j*th local mode is expressed as follows:

(30) $$\begin{array}{cc} S_{1}\left(\rho_{j}\right)= & -\left(1-f\tanh^{2}r\right)\ln\left(1-f\tanh^{2}r\right)\\ \; & -f\tanh^{2}r\left\{\ln\left(f{\mathrm{sech}}^{2}r\right)+\cosh^{2}r\ln\left(\tanh^{2}r\right)\right\}, \end{array}$$

with j∈{1,2} and the von Neumann entropy of the global state ρ is expressed as follows:

(31) $$S\left(\rho \right)=-\lambda_{+}\ln\lambda_{+}-\lambda_{-}\ln\lambda_{-},$$

with $\lambda_{\pm }=\frac{1}{2}\left\{1\pm \sqrt{1-4f\left(1-f\right)\tanh^{2}r}\right\}$. We have the following expression:

(32) $$S_{2}\left(\rho_{1}\right)=S_{2}\left(\rho_{2}\right)=-\ln\left(1-2f\tanh^{2}r+\frac{2f^{2}\tanh^{4}r}{1+\tanh^{2}r}\right),$$

and $S_{2}\left(\rho \right)=-\ln\left(\lambda_{+}^{2}+\lambda_{-}^{2}\right)$.

The joint quadrature distribution of the CV Werner state is expressed as follows:

(33) $$P_{\rho }^{\left\{\phi_{1},\phi_{2}\right\}}\left(q_{1},q_{2}\right)=fP_{|\psi_{r}\rangle \langle \psi_{r}|}^{\left\{\phi_{1},\phi_{2}\right\}}\left(q_{1},q_{2}\right)+\frac{1-f}{\pi }\exp\left(-q_{1}^{2}-q_{2}^{2}\right),$$

where $P_{|\psi_{r}\rangle \langle \psi_{r}|}^{\left\{\phi_{1},\phi_{2}\right\}}\left(q_{1},q_{2}\right)$ is given by

(34) $$P_{|\psi_{r}\rangle \langle \psi_{r}|}^{\left\{\phi_{1},\phi_{2}\right\}}\left(q_{1},q_{2}\right)=\frac{1}{2\pi \sqrt{u^{2}-v^{2}}}\exp\left[-\frac{u\left(q_{1}^{2}+q_{2}^{2}\right)-2vq_{1}q_{2}}{2(u^{2}-v^{2})}\right],$$

with $u=\frac{1}{2}\cosh2r$ and $v=\frac{1}{2}\sinh2r\cos\left(\phi_{1}+\phi_{2}\right)$. We numerically determine that the classical mutual information extractable by joint homodyne detection $C_{\mathrm{HD}}^{\left\{\phi_{1},\phi_{2}\right\}}\left(\rho \right)$ is maximized when $\phi_{1}+\phi_{2}=0$, just like the squeezed thermal states and the pair coherent states in Section 3.1 and Section 3.2, respectively.

Let us briefly explain the commonality of the examples. All the examples have symmetrical number correlations and real density matrix elements, i.e., $\rho =\sum_{n,m}\rho_{n,m}\left|n\rangle \langle m\right|⊗\left|n\rangle \langle m\right|$ and $\rho_{n,m}=\rho_{n,m}^{*}$ for all *n* and *m*. These features yield strong position–position correlation ($\phi_{1}=\phi_{2}=0$) and momentum–momentum correlation ($\phi_{1}=\phi_{2}=\frac{\pi }{2}$) in general, which are reflected in the intermodal correlation terms of the covariance matrix. In addition, the effect of the rotation $\phi_{1}$ in the first mode can be canceled out by the counter-rotation $\phi_{2}=-\phi_{1}$ in the second mode because of the symmetry in the density matrix ρ, i.e., $e^{i{\hat{n}}_{1}\phi_{1}}e^{i{\hat{n}}_{2}\phi_{2}}\rho e^{-i{\hat{n}}_{2}\phi_{2}}e^{-i{\hat{n}}_{1}\phi_{1}}=\sum_{n,m}\rho_{n,m}e^{i\left(n-m\right)\left(\phi_{1}+\phi_{2}\right)}\left|n\rangle \langle m\right|⊗\left|n\rangle \langle m\right|$. Therefore, it may be natural that $C_{\mathrm{HD}}^{\left\{\phi_{1},\phi_{2}\right\}}\left(\rho \right)$ is maximized when $\phi_{1}+\phi_{2}=0$ not only for Gaussian states but also for some non-Gaussian states.

**Figure 3:**
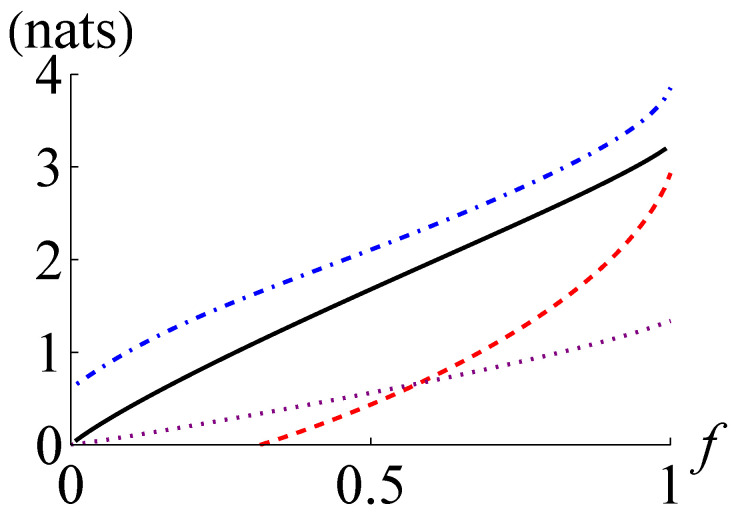
Quantum mutual information I(ρ) (black solid line), its lower and upper bounds $I_{-}\left(\rho \right)$ (red dashed line), and $I_{+}\left(\rho \right)$ (blue dot-dashed line), respectively, and the maximum of the classical mutual information extractable by joint homodyne detection $C_{\mathrm{HD}}\left(\rho \right)$ (purple dotted line) for the CV Werner state $\rho =f|\psi_{r}\rangle \langle \psi_{r}\left|+\left(1-f\right)|0\rangle \langle 0|⊗|0\rangle \langle 0\right|$ with r=1 against the fraction *f*.

In Figure 3, we plot I(ρ), $I_{+}\left(\rho \right)$, $I_{-}\left(\rho \right)$, and $C_{\mathrm{HD}}\left(\rho \right)$ for a CV Werner state $\rho =f|\psi_{r}\rangle \langle \psi_{r}\left|+\left(1-f\right)|0\rangle \langle 0|⊗|0\rangle \langle 0\right|$ with r=1. The curves in Figure 3 reveal that $I_{-}\left(\rho \right)$ becomes positive for f>0.316 and surpasses $C_{\mathrm{HD}}\left(\rho \right)$ for f>0.580. The CV Werner state is Gaussian for f∈{0,1} and non-Gaussian for 0<f<1. The ratio of $I_{-}\left(\rho \right)$ to I(ρ) has the maximum of about 0.905 at f=1 and the ratio of $I_{+}\left(\rho \right)$ to I(ρ) has the minimum of about 1.140 at f≃0.88. This observation also supports that $I_{-}\left(\rho \right)$ and $I_{+}\left(\rho \right)$ may generally work better for the quantum states with weaker non-Gaussianity and larger quantum mutual information.

## 4. Bounds for Quantum Total Correlation

Quantum mutual information was extended to the multimode case [19,31] as follows:

(35) $$\begin{array}{cc} T(\rho_{12\cdots N}) & =S(\rho_{12\cdots N}||\rho_{1}⊗\rho_{2}⊗\cdots ⊗\rho_{N})\\ \; & =\sum_{j=1}^{N}S\left(\rho_{j}\right)-S\left(\rho_{12\cdots N}\right), \end{array}$$

which becomes identical to Equation (1) when N=2. We denote an *N*-mode global quantum state $\rho_{12\cdots N}$ as ρ for simplicity.

The upper and lower bounds on the quantum total correlation of an *N*-mode quantum state ρ are given by

(36) $$T_{+}\left(\rho \right)\equiv T\left(\rho_{G}\right)+F\left(\rho \right),$$

and

(37) $$T_{-}\left(\rho \right)\equiv T\left(\rho_{G}\right)-\sum_{j=1}^{N}F\left(\rho_{j}\right)+\max_{k}F\left(\rho_{k}\right),$$

respectively. Note that Equations (36) and (37) become Equations (10) and (11), respectively, for N=2.

### 4.1. Gaussian State

For every *N*-mode Gaussian state ρ, $T_{-}\left(\rho \right)$ is smaller than T(ρ) by (N−1)ln(e/2),

(38) $$T\left(\rho \right)-T_{-}\left(\rho \right)=\left(N-1\right)\ln\frac{e}{2},$$

and $T_{+}\left(\rho \right)$ is greater than T(ρ) by Nln(e/2),

(39) $$T_{+}\left(\rho \right)-T\left(\rho \right)=N\ln\frac{e}{2},$$

which reveal that $T_{-}\left(\rho \right)$ and $T_{+}\left(\rho \right)$ work well for a broad range of Gaussian states with small differences.

### 4.2. Entangled Coherent State

We here investigate an entangled coherent state in the form of

(40) $$|\Psi_{M}\rangle =\frac{1}{\sqrt{N_{-}\left(\sqrt{M}\gamma \right)}}{(|\gamma \rangle }_{1}{|\gamma \rangle }_{2}{\cdots |\gamma \rangle }_{M}{-|-\gamma \rangle }_{1}{|-\gamma \rangle }_{2}{\cdots |-\gamma \rangle }_{M}),$$

where $|\gamma \rangle =\exp\left(-\frac{{\left|\gamma \right|}^{2}}{2}\right)\sum_{k=0}^{\infty }\frac{\gamma^{k}}{\sqrt{k!}}|k\rangle$ denotes a coherent state with complex amplitude γ and $N_{\pm }\left(x\right)={2\pm 2\exp(-2|x|}^{2})$ is related to the normalization factor. We set γ as real for simplicity. The covariance matrix of $|\Psi_{M}\rangle$ is expressed as follows:

(41) $$\Gamma_{|\Psi_{M}\rangle \langle \Psi_{M}|}=\left(\begin{array}{cccc} X & Y & \cdots & Y\\ Y & X & \cdots & Y\\ \vdots & \vdots & \ddots & \vdots \\ Y & Y & \cdots & X \end{array}\right),$$

where the 2×2 block matrice X and Y are expressed by $X=\mathrm{diag}\left(X_{11},X_{22}\right)=\mathrm{diag}\left(\frac{1}{2}+\gamma^{2}\left\{\coth\left(M\gamma^{2}\right)+1\right\},\frac{1}{2}+\gamma^{2}\left\{\coth\left(M\gamma^{2}\right)-1\right\}\right)$ and $Y=\mathrm{diag}\left(Y_{11},Y_{22}\right)=\mathrm{diag}\left(X_{11}-\frac{1}{2},X_{22}-\frac{1}{2}\right)$, respectively.

For the covariance matrix in Equation (41), one of the symplectic eigenvalues is expressed as follows:

(42) $$\Lambda =\sqrt{\left\{X_{11}+\left(M-1\right)Y_{11}\right\}\left\{X_{22}+\left(M-1\right)Y_{22}\right\}},$$

and the other ones are identical to $\frac{1}{2}$. This result is due to the fact the entangled coherent state can be generated from an odd cat state and M−1 vacuum states using a beam-splitter network [32]:

(43) $$|\Psi_{M}\rangle ={\hat{B}}_{M}\frac{1}{\sqrt{N_{-}\left(\sqrt{M}\gamma \right)}}{(|\sqrt{M}\gamma \rangle }_{1}-|-\sqrt{M}{\gamma \rangle }_{1}{)|0\rangle }_{2}\cdots {|0\rangle }_{M},$$

where the *M*-mode unitary operation ${\hat{B}}_{M}$ by a beam-splitter network transforms a coherent state and M−1 vacuum states as

(44) $${\hat{B}}_{M}{|\gamma \rangle }_{1}{|0\rangle }_{2}{\cdots |0\rangle }_{M}=|\frac{1}{\sqrt{M}}{\gamma \rangle }_{1}|\frac{1}{\sqrt{M}}{\gamma \rangle }_{2}\cdots {|\frac{1}{\sqrt{M}}\gamma \rangle }_{M}.$$

The symplectic eigenvalues of the covarinace matrix $\Gamma_{\rho }$ of a quantum state ρ are invariant under Gaussian unitary operations. Therefore, the symplectic eigenvalue Λ in Equation (42) and the other ones are originated from the odd cat state and vacuum states, respectively.

The local state for the *j*th mode is expressed as follows:

(45) $$\begin{array}{cc} \rho_{j}= & \frac{1}{N_{-}\left(\sqrt{M}\gamma \right)}{[|\gamma \rangle \langle \gamma |}_{j}{+|-\gamma \rangle \langle -\gamma |}_{j}-e^{-2\left(M-1\right)\gamma^{2}}{(|\gamma \rangle \langle -\gamma |}_{j}+|-\gamma \rangle \langle \gamma {|}_{j})], \end{array}$$

which can be recasted as follows:

(46) $$\begin{array}{c} \rho_{j}=\tilde{f}{\left|-;\gamma \rangle \langle -;\gamma \right|}_{j}+\left(1-\tilde{f}\right){\left|+;\gamma \rangle \langle +;\gamma \right|}_{j}, \end{array}$$

where the even and odd cat states, i.e., $|\pm ;\gamma \rangle =\frac{1}{\sqrt{N_{\pm }\left(\gamma \right)}}(|\gamma \rangle \pm |-\gamma \rangle )$, form an orthonormal basis and the fraction of the odd cat state is given by $\tilde{f}=\frac{N_{-}\left(\gamma \right)N_{+}\left(\sqrt{M-1}\gamma \right)}{4N_{-}\left(\sqrt{M}\gamma \right)}$.

The quantum total correlations of the global state $\rho =|\Psi_{M}\rangle \langle \Psi_{M}|$ and its Gaussian reference $\rho_{G}$ are obtained as follows:

(47) $$T\left(\rho \right)=-M\{\tilde{f}\ln\tilde{f}+\left(1-\tilde{f}\right)\ln\left(1-\tilde{f}\right)\},$$

and

(48) $$T\left(\rho_{G}\right)=Mg\left(\sqrt{X_{11}X_{22}}\right)-g\left(\Lambda \right),$$

respectively. Furthermore, the quantum Rényi-2 entropies for the local state for the *j*th mode $\rho_{j}$ and its Gaussian reference $\rho_{j,G}$ are expressed as follows:

(49) $$S_{2}\left(\rho_{j}\right)=-\ln\left\{{\tilde{f}}^{2}+{\left(1-\tilde{f}\right)}^{2}\right\},$$

and

(50) $$S_{2}\left(\rho_{j,G}\right)=-\ln\left(2\sqrt{X_{11}X_{22}}\right),$$

respectively, with j∈{1,2,…,M}.

**Figure 4:**
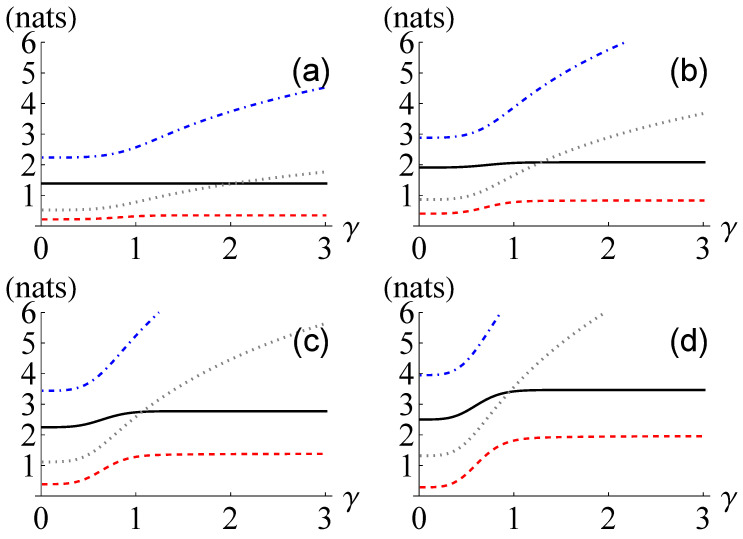
Quantum total correlation T(ρ) (black solid line), its lower and upper bounds $T_{-}\left(\rho \right)$ (red dashed line), and $T_{+}\left(\rho \right)$ (blue dot-dashed line), respectively, and the quantum total correlation of the reference Gaussian state $T(\rho_{G})$ (gray dotted line) for *M*-mode entangled coherent states $\rho =|\Psi_{M}\rangle \langle \Psi_{M}|$ with (**a**) M=2, (**b**) M=3, (**c**) M=4, and (**d**) M=5 against the coherent amplitude γ.

In Figure 4, we plot T(ρ), $T(\rho_{G})$, $T_{-}\left(\rho \right)$ and $T_{+}\left(\rho \right)$ for M={2,3,4,5}. As the overlap between the coherent states |γ〉 and |−γ〉, i.e., $\langle \gamma |-\gamma \rangle =\exp\left(-2\gamma^{2}\right)$, becomes negligible when the coherent amplitude γ is sufficiently large, the entangled coherent state behaves like a Greenberger–Horne–Zeilinger state, e.g., $\frac{1}{\sqrt{2}}{(|+\rangle }_{1}{\cdots |+\rangle }_{M}{-|-\rangle }_{1}{\cdots |-\rangle }_{M})$ with 〈+|−〉=0. Therefore, the quantum total correlation T(ρ) converges to Mln2 as γ increasing. By contrast, the quantum total correlation of the reference Gaussian state $T(\rho_{G})$ diverges to infinity as γ increasing. This phenomenon confirms that the quantum total correlation does not exhibit Gaussian extremality in general [18]. Furthermore, the maximum ratio between T(ρ) and the lower bound $T_{-}\left(\rho \right)$ becomes greater as the number of modes increases. Notably, we also observe similar behaviors for an entangled coherent state in the form of

(51) $$|{\tilde{\Psi }}_{M}\rangle =\frac{1}{\sqrt{N_{+}\left(\sqrt{M}\gamma \right)}}{(|\gamma \rangle }_{1}{|\gamma \rangle }_{2}{\cdots |\gamma \rangle }_{M}{+|-\gamma \rangle }_{1}{|-\gamma \rangle }_{2}{\cdots |-\gamma \rangle }_{M}),$$

which can be generated from an even cat state by using a beam-splitter network. It suggests that the proposed lower bound can be feasible for estimating the quantum total correlation of multimode quantum states.

## 5. Bounds for Quantum Conditional Entropy

The quantum conditional entropy for a two-mode quantum state ρ is defined as follows:

(52) $$S{\left(1|2\right)}_{\rho }=S\left(\rho \right)-S\left(\rho_{2}\right),$$

which becomes non-negative for every separable state [33]. If $S{\left(1|2\right)}_{\rho }<0$, it witnesses that ρ is entangled. Note that we can reformulate the quantum mutual information by using the quantum conditional entropy as follows:

(53) $$I\left(\rho \right)=S\left(\rho_{1}\right)-S{\left(1|2\right)}_{\rho }.$$

We introduce the upper and lower bounds for the quantum conditional entropy as follows:

(54) $$S_{+}{\left(1|2\right)}_{\rho }=S{\left(1|2\right)}_{\rho_{G}},$$

and

(55) $$S_{-}{\left(1|2\right)}_{\rho }=S{\left(1|2\right)}_{\rho_{G}}-F\left(\rho \right),$$

respectively. It is known that $S{\left(1|2\right)}_{\rho }$ is upper-bounded by $S_{+}{\left(1|2\right)}_{\rho }=S{\left(1|2\right)}_{\rho_{G}}$ [14]. We derive Equation (55) as follows:

(56) $$\begin{array}{cc} S{\left(1|2\right)}_{\rho } & =S\left(\rho \right)-S\left(\rho_{2}\right)\\ \; & \geq S\left(\rho \right)-S\left(\rho_{2,G}\right)\\ \; & =\left\{S\left(\rho \right)-S\left(\rho_{G}\right)\right\}+S{\left(1|2\right)}_{\rho_{G}}\\ \; & \geq S{\left(1|2\right)}_{\rho_{G}}-F\left(\rho \right)\\ \; & =S_{-}{\left(1|2\right)}_{\rho }. \end{array}$$

We observe that $S{\left(1|2\right)}_{\rho }\leq S_{+}{\left(1|2\right)}_{\rho }$ and $S{\left(1|2\right)}_{\rho }\geq S_{-}{\left(1|2\right)}_{\rho }$ are sufficient but not necessary conditions for $I\left(\rho \right)\geq I_{-}\left(\rho \right)$ and $I\left(\rho \right)\leq I_{+}\left(\rho \right)$, respectively. Note that we can reformulate Equations (14) and (16) as follows:

(57) $$\begin{array}{cc} I(\rho ) & =S\left(\rho_{1}\right)-S{\left(1|2\right)}_{\rho }\\ \; & \leq S\left(\rho_{1}\right)-S_{-}{\left(1|2\right)}_{\rho }\\ \; & \leq S\left(\rho_{1,G}\right)-S{\left(1|2\right)}_{\rho_{G}}+F\left(\rho \right)\\ \; & =I\left(\rho_{G}\right)+F\left(\rho \right)\\ \; & =I_{+}\left(\rho \right), \end{array}$$

and

(58) $$\begin{array}{cc} I(\rho ) & =S\left(\rho_{1}\right)+S\left(\rho_{2}\right)-S\left(\rho \right)\\ \; & \geq \max_{j,k\in \{1,2\}}\left\{S\left(\rho_{j}\right)-S_{+}{\left(j|k\neq j\right)}_{\rho }\right\}\\ \; & \geq \max_{j,k\in \{1,2\}}\left\{S\left(\rho_{j,G}\right)-F\left(\rho_{j}\right)-S{\left(j|k\neq j\right)}_{\rho_{G}}\right\}\\ \; & =I\left(\rho_{G}\right)-\min_{j\in \{1,2\}}F\left(\rho_{j}\right)\\ \; & =I_{-}\left(\rho \right), \end{array}$$

respectively. If we attempt to derive the upper and lower bounds for the quantum condition entropy by using $S{\left(1|2\right)}_{\rho }=S\left(\rho_{1}\right)-I\left(\rho_{12}\right)$ and the upper and lower bounds for the quantum mutual information, i.e., $I_{+}\left(\rho_{12}\right)$ and $I_{-}\left(\rho_{12}\right)$, we only get some weaker bounds.

### 5.1. Gaussian State

For every two-mode Gaussian state ρ, $S_{+}{\left(1|2\right)}_{\rho }$ is equal to $S{\left(1|2\right)}_{\rho }$ and $S_{-}{\left(1|2\right)}_{\rho }$ is smaller than $S{\left(1|2\right)}_{\rho }$ by 2ln(e/2),

(59) $$S{\left(1|2\right)}_{\rho }-S_{-}{\left(1|2\right)}_{\rho }=2\ln\frac{e}{2},$$

which reveal that $S_{+}{\left(1|2\right)}_{\rho }$ and $S_{-}{\left(1|2\right)}_{\rho }$ work well for a broad range of Gaussian states with small differences.

### 5.2. CV Werner State

For the CV Werner state introduced in Section 3.3, the quantum conditional entropy is obtained by the following expression:

(60) $$S{\left(1|2\right)}_{\rho }=S\left(\rho \right)-S\left(\rho_{2}\right),$$

where S(ρ) and $S(\rho_{2})$ are given by Equations (30) and (31), respectively. We numerically observe that the quantum conditional entropy of the CV Werner state is always non-positive. By constrast, if we look into a mixture of vacuum and a coherent state in the form of $\rho =f|\gamma_{1}\rangle \langle \gamma_{1}\left|⊗\right|\gamma_{2}\rangle \langle \gamma_{2}\left|+\left(1-f\right)|0\rangle \langle 0|⊗|0\rangle \langle 0\right|$, its quantum conditional entropy is always non-negative. This observation clearly manifests that the two-mode squeezed vacuum plays a dominant role in the nonclassical behaviors of the CV Werner state.

**Figure 5:**
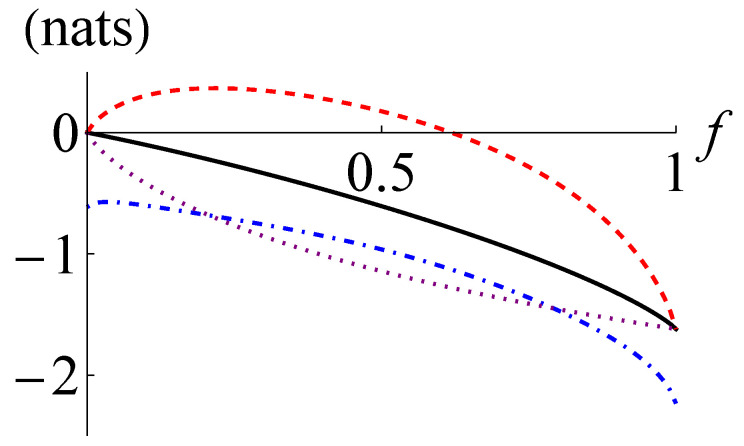
Quantum conditional entropy $S{\left(1|2\right)}_{\rho }$ (black solid line), its upper and lower bounds $S_{+}{\left(1|2\right)}_{\rho }$ (red dashed line), and $S_{-}{\left(1|2\right)}_{\rho }$ (blue dot-dashed line), respectively, and $-S(\rho_{2,G})$ (purple dotted line) for the CV Werner state $\rho =f|\psi_{r}\rangle \langle \psi_{r}\left|+\left(1-f\right)|0\rangle \langle 0|⊗|0\rangle \langle 0\right|$ against the fraction *f*.

In Figure 5, we plot $S{\left(1|2\right)}_{\rho }$, $S_{-}{\left(1|2\right)}_{\rho }$, $S_{+}{\left(1|2\right)}_{\rho }$ and $-S(\rho_{2,G})$ for the CV Werner state with r=1. We observe that $S_{+}{\left(1|2\right)}_{\rho }$ witnesses the entaglement of the CV Werner state for f>0.618. Furthermore, we find that $S_{-}{\left(1|2\right)}_{\rho }$ outperforms a simple lower bound of the conditional entropy, i.e., $-S(\rho_{2,G})$, for 0.208<f<0.792. The ratio of $S_{+}{\left(1|2\right)}_{\rho }$ to $S{\left(1|2\right)}_{\rho }$ becomes unity at f=1 and the ratio of $S_{-}{\left(1|2\right)}_{\rho }$ to $S{\left(1|2\right)}_{\rho }$ has the minimum of about 1.301 at f≃0.905. This observation suggests that $S_{+}{\left(1|2\right)}_{\rho }$ and $S_{-}{\left(1|2\right)}_{\rho }$ may generally work better for the quantum states with weaker non-Gaussianity and larger absolute value of quantum conditional entropy.

## 6. Concluding Remarks

We have proposed upper and lower bounds for the quantum mutual information of CV quantum states. The bounds are expressed by functions of the purities and the symplectic eigenvalues of the covariance matrix, which are accessible without reconstructing the multimode quantum states. In [34], quantum mutual information has been extended by using sandwiched quantum Rényi-α relative entropy [35,36]. The sandwiched quantum Rényi-α relative entropy of τ with respect to σ is defined as ${\tilde{S}}_{\alpha }\left(\tau ||\sigma \right)=\frac{1}{\alpha -1}\ln\mathrm{tr}\left[{\left(\sigma^{\frac{1-\alpha }{2\alpha }}\tau \sigma^{\frac{1-\alpha }{2\alpha }}\right)}^{\alpha }\right]$, which becomes the quantum relative entropy S(τ||σ) in the limit of α→1 and has an ordering relation as ${\tilde{S}}_{\alpha^{\prime }}\left(\rho ||\sigma \right)\geq {\tilde{S}}_{\alpha }\left(\rho ||\sigma \right)$ for $\alpha^{\prime }>\alpha$. In general, it is much more difficult to determine the quantum Rényi-α mutual information $I_{\alpha }\left(\rho \right)={\tilde{S}}_{\alpha }\left(\rho |\right|\rho_{1}⊗\rho_{2})$ compared to the quantum mutual information I(ρ). Although the quantum mutual information I(ρ) can be repesented by a function of von Neumann entropy for local and global quantum states as $I\left(\rho \right)=S\left(\rho_{1}\right)+S\left(\rho_{2}\right)-S\left(\rho \right)$, the quantum Rényi-α mutual information $I_{\alpha }\left(\rho \right)$ with α≠1 cannot be represented by a function of quantum Rényi-α entropy of local and global quantum states, e.g., $I_{\alpha }\left(\rho \right)\neq S_{\alpha }\left(\rho_{1}\right)+S_{\alpha }\left(\rho_{2}\right)-S_{\alpha }\left(\rho \right)$. Notably, the proposed bounds $I_{+}\left(\rho \right)$ and $I_{-}\left(\rho \right)$ also work for the Rényi-α mutual information $I_{\alpha }\left(\rho \right)$ with α≤1 and α≥1, respectively, because of the ordering relation.

Furthermore, we have extended our method to the quantum total correlation of multimode CV quantum states. We also have investigated how the bounds for the quantum mutual information are related to the bounds for the quantum conditional entropy. Notably, the quantum mutual information can be generalized to a multimode situation by using another method [37,38]. In addition, several methods have been proposed to quantify the genuine multimode correlations by information-theoretic quantities [39,40]. We hope that our approach will be extended to such elaborate measures.

## Data Availability

Not applicable.

## Abbreviations

The following abbreviations are used in this manuscript:

CV	Continuous-Variable
POVM	Positive Operator-Valued Measure
